# Randomized controlled trial on eurythmy therapy versus slow-paced physical exercises for the treatment of fatigue in metastatic breast cancer patients

**DOI:** 10.1093/oncolo/oyaf343

**Published:** 2025-10-22

**Authors:** Eliane Timm, Ilana Berlowitz, Ursula Wolf

**Affiliations:** Institute of Complementary and Integrative Medicine, Faculty of Medicine, University of Bern, Bern 3012, Switzerland; Institute of Complementary and Integrative Medicine, Faculty of Medicine, University of Bern, Bern 3012, Switzerland; Institute of Complementary and Integrative Medicine, Faculty of Medicine, University of Bern, Bern 3012, Switzerland

**Keywords:** cancer-related fatigue, metastatic breast cancer, eurythmy therapy, mindful movement therapy, randomized controlled trial

## Abstract

**Background:**

Cancer-related fatigue is among the most taxing symptoms of breast cancer patients, but there is currently no established treatment standard. This study assessed the effect of eurythmy therapy (ERYT), a tailored mindful movement therapy, compared to slow-paced physical exercises (CoordiFit) on fatigue in metastatic breast cancer patients.

**Methods:**

We used a randomized controlled, open-label, multi-center, two-arm design. Ten certified oncology centers across Switzerland participated in the trial. Women with metastatic breast cancer who agreed to participate in the study were randomly allocated to one of the two study arms in a 1:1 ratio. The intervention sessions in both groups followed the same schedule in terms of frequency and duration over a period of 20 weeks. Outcomes were assessed using standard clinical assessment scales at baseline, weeks 8, 14, and 20, as well as at 6- and 12-months follow-up. The primary endpoint was the change in cancer-related fatigue (baseline to end of intervention), whereas secondary endpoints included quality of life, pain, sleep quality, depression, anxiety, distress, and arm mobility. Data was analyzed using two-way mixed ANOVA.

**Results:**

The study was terminated before its completion due to insufficient enrollment. Prior to its closure, a total of 19 patients (median age: 59.5, range 51-82) agreed to participate in the study, of whom 10 completed the full intervention (*n *= 5 per group). Although the small sample size limits the possibility of statistical inference, tentative analyses pointed to significant improvements in fatigue in response to both experimental and control interventions (*F*(1,8)=14.176, *P* = .006), with no difference between groups. Among secondary endpoints we found a main group effect in quality of life, which was significantly higher in the ERYT group (*F*(1,8)=7.179, *P* = .028), as well as an interaction effect in pain interference, the latter significantly improving in the ERYT group but not the CoordiFit group (*F*(1,8)=7.977, *P* = .022).

**Conclusion:**

While the results show a promising decrease in cancer-related fatigue in response to ERYT, this held true also for the movement-based control intervention, suggesting ERYT to be valuable, but not superior to other movement-based approaches for cancer-related fatigue. However, the small sample size limits the conclusions that can be drawn.

**ClinicalTrials.gov identifier:**

#NCT04024267

Lessons Learned

- Early trial closure due to insufficient recruitment.
- The study suggests that both eurythmy therapy as well as slow-paced physical exercise may equally reduce cancer-related fatigue, with no indications for superiority of either. However, significant recruitment barriers led to a low rate of enrollment and sample size, which limits the capacity for conclusive results.
- The lack of accrual was linked to challenges in recruitment due to the COVID-19 pandemic (increased demands on healthcare staff, restricted hospital access, social contact restrictions, etc.), as well as, presumably, to a low per patient fee (compared to usual patient fees in clinical trials), which may have impacted center incentivation for recruitment.

## Trial information

**Table:** 

Trial information	
**Disease**	Metastatic breast cancer in women
**Stage of disease/Treatment**	Any stage of metastatic breast cancer
**Type of study**	Randomized controlled open-label two-arm multi-center trial.
**Primary endpoints**	Cancer-related fatigue: change from baseline to end of intervention (20 weeks).
**Secondary endpoints**	Quality of life, pain, sleep quality, depression, anxiety, distress, arm mobility: changes from baseline to end of intervention (20 weeks).
**Additional details of endpoints or study design** The study (October 2019-July 2025) experienced significant recruitment challenges in part due to the COVID-19 pandemic. The first participant was recruited in January 2021 and the last one in February 2024, after which recruitment was halted. All endpoints were assessed at 6 measurement points, namely at week 0 (baseline), 8, 14, 20 (completion of the intervention), 6 months after completion (6-months follow-up), and 12 months after completion (12-months follow-up), except for depression and anxiety, which were measured only at baseline, intervention completion, and at both follow-ups. We additionally assessed retention and compliance.	

## Intervention information

**Table:** 

Study interventions	
Name	Eurythmy therapy (experimental group)
**Type**	Mindful movement therapy
**Detailed description**	ERYT is a mindful movement therapy that involves the performance of integrated sequences of body movements related to speech sounds in a state of focused concentration and intentionality.^1–5^ Preliminary evidence suggests physiological and mental health benefits.^6–17^ ERYT exercises are tailored to specific medical conditions or symptoms,^5^ such as the so-called “cancer series” designed for breast cancer patients (sometimes also called OEMLIBD based on the sound sequence it involves),^5^ ^,^ ^18^ which was used in the current study. The experimental intervention consisted of a total of 13 ERYT sessions administered by a certified therapist. The sessions were held individually or in groups of up to four participants. The exercise sequence followed a defined progression, generally starting with basic exercises and gradually moving towards more advanced ones, all the while considering individual capacities and limitations.
	
**Name**	**CoordiFit (control group)**
**Type**	Slow-paced physical exercises
**Detailed description**	The CoordiFit control intervention consisted of structured slow-movement exercises that involved coordination, stability, balance, and dexterity. They were designed as a control intervention requiring a similar level of effort as the ERYT exercises, but without being tailored to cancer-related fatigue and with no mindfulness component. The exercises included simple movement patterns to mobilize the shoulders, arms, legs, and spine (eg, rolling the head forward and down toward the feet, vertebra by vertebra), as well as balance and coordination exercises. Like with the ERYT exercises, the 13 CoordiFit sessions were held individually or in groups of up to four participants and were administered by a certified physiotherapist.
**Schedule of Administration** For both ERYT and CoordiFit groups the overall duration of the intervention was 20 weeks. It involved a total of thirteen 45-min training sessions (either ERYT or CoordiFit), which were scheduled once a week during the first six weeks of the trial, and every other week during the remaining time. Furthermore, for the duration of the 20 weeks of the intervention, patients were instructed to practice the exercises at home for about 15 min per day, if possible six times per week.	

## Patient characteristics

**Table:** 

Patient characteristics at baseline	
**Number of patients, female**	19
**Number of patients, male**	-
**Stage**	III-IV
**Age in years: median (range)**	59.5 (51-82)
**Performance status: ECOG 0 or 1**	19
**Performance status: ECOG 2 or above**	0
**Cancer types or histologic subtypes**	Metastatic breast cancer
**Menopausal status, *n *= 18**	
** Pre or peri-menopausal**	2
** Post-menopausal**	16
**BMI, mean (*n *= 16)**	27.45
**Initial receptor status, *n *= 18**	
** HER2**	
** Negative**	11
** Positive**	7
** Progesterone**	
** Negative**	3
** Positive**	15
** Estrogen**	
** Negative**	1
** Positive**	17
**Stage of disease, *n *= 18**	
** Initial diagnosis with metastases**	6
** Metastases only in the course of treatment**	8
** Recurrence with metastases**	4
**In palliative treatment, *n *= 18**	16
**Number of participants that received the following types of therapies, *n *= 18**	
** Any type of surgery**	17
** Tumorectomy**	12
** Mastectomy**	9
** Breast reconstruction**	6
** Removement of axillary lymph nodes**	11
** Chemotherapy**	13
** 1 chemotherapy**	5
** 2 chemotherapies**	5
** 3 chemotherapies**	1
** 4 chemotherapies**	1
** 5 chemotherapies**	0
** 6 chemotherapies**	1
** Radiotherapy (any body part)**	15
** 1 radiotherapy**	11
** 2 radiotherapies**	2
** 3 radiotherapies**	0
** 4 radiotherapies**	2
** Breast**	13
** 1 radiotherapy**	12
** 2 radiotherapies**	1
** Other locations**	7
** 1 radiotherapy**	5
** 2 radiotherapies**	1
** 3 radiotherapies**	1
** HER2 antibody therapy**	0
** Endocrine therapy**	6
**Patients on active treatment, *n *= 18**	
** Chemotherapy**	6
** Radiotherapy**	0
** HER2 antibody therapy**	4
** Endocrine therapy**	9
**Location of metastases, *n *= 18**	
** Liver**	4
** Brain**	2
** Bones**	12
** Lymph nodes**	11
** Lung, pleura**	3
** Heart, mediastinum**	1
** Other**	2
**Laboratory (means)**	
** Hemoglobin (g/dL), *n *= 18**	12.5
** TSH basal (mU/L), *n *= 7**	1.49
** FT3 (pmol/L), *n *= 5**	4.47
** FT4 (pmol/L), *n *= 5**	12.84
** AST (U/L), *n *= 15**	25.6
** ALT (U/L), *n *= 15**	22.34
** Gamma GT (U/L), *n *= 16**	73.8 30.43 if one outlier (GGT: 681U/L) removed
**Number of comorbidities, *n *= 18**	
** Thyroid dysfunction**	2
** Liver dysfunction**	2
** Bronchial asthma**	1
** Sleep apnea**	0
** Restless legs syndrome**	1
** Depressive mood/depression**	5
**Therapies against fatigue, *n *= 18**	
** Medication**	0
** Psychosocial methods**	2
** Therapeutic massage**	2
** Acupuncture**	0
** Nutrition counseling**	0
** Mistletoe injections**	5
** Physical exercise**	6
** Mindfulness-based exercises**	4
** Other**	1
**Patient characteristics notes:** Trial inclusion criteria: Female aged ≥18 years. Ability to provide informed consent. Functional Assessment of Chronic Illness Therapy—Fatigue subscale score (FACIT-F) < 34. Histologically or cytologically confirmed metastatic breast cancer. Eastern Cooperative Oncology Group (ECOG) grade 0 or 1. Ability to physically and cognitively perform an active movement therapy. Ability to read, write, and speak German, French, or Italian. Exclusion criteria: Limitations or contraindication that could hinder the participation in the interventions or long-term follow-up. Psychiatric, addictive or other disorders that prevents the patient from adhering to the protocol requirements. Significant uncontrolled cardiac disease. Hemoglobin < 9 g/dL.	

## Primary assessment method

**Table:** 

Primary assessment method	Overall	Intervention	Control
**Number of patients enrolled**	19	11	8
**Number of intervention completers**	10	5	5
**Evaluation method**	Descriptive statistics, two-way mixed ANOVA. The originally envisaged statistical analysis plan was inadequate due to the small number of trial participants; the original protocol^19^ was hence slightly modified to accommodate the changed circumstances.		
**Outcome notes:** For retention and compliance see flow chart in Figure 1. Descriptive statistics of all endpoints can be found in Table 1. For cancer-related fatigue change scores across the measurement points see Figure 2. Changes from baseline to intervention completion and results of the two-way mixed ANOVAs are shown in Table 1. Figure 3 shows a waterfall plot for intervention completers. Measures: Cancer-related fatigue: Functional Assessment of Chronic Illness Therapy—Fatigue subscale (FACIT-F)^20^; Quality of life: Functional Assessment of Cancer Therapy—General (FACT-G)^21^^,^^22^; Sleep Quality: Pittsburgh Sleep Quality Index (PSQI)^23^^,^^24^; Pain: Brief Pain Inventory (BPI)^25^^,^^26^; Depression: Patient Health Questionnaire-9 (PHQ-9)^27–29^; Anxiety: General Anxiety Disorder-7 (GAD-7)^27–29^; Distress: National Comprehensive Cancer Network Distress Thermometer^30^; Arm mobility: Rating (0—not moveable, 10—very good moveable); Retention: number, time to, and causes of attrition; Compliance: number of sessions attended and weekly hours spent with home practice as per diary forms filled in by patients.			

**Table 1.** Primary and secondary endpoints: means and standard deviations of all study participants.

	Baseline	Week 8	Week 14	End (week 20)	6 months follow-up	12 month follow-up	Change scores: end minus baseline scores
**Primary endpoint**							
** Fatigue^1^**	21.37 (7.05) *n* = 19	32.67 (7.13) *n* = 12	34.75 (9.20) *n* = 12	36.8 (6.65) *n* = 10	40.13 (6.45) *n* = 8	39.88 (8.76) *n* = 8	15.43*^a^ *n* = 10
** ERYT**	23 (5.48) *n* = 11	33.57 (8.16) *n* = 7	35.57 (9.81) *n* = 7	40.20 (4.44) *n* = 5	42.40 (6.19) *n* = 5	42.00 (10.30) *n* = 5	17.20 *n* = 5
** Coord**	19.13 (8.66) *n* = 8	31.40 (6.02) *n* = 5	33.6 (9.24) *n* = 5	33.40 (7.13) *n* = 5	36.33 (5.86) *n* = 3	36.34 (5.13) *n* = 3	14.28 *n* = 5
**Secondary endpoints**							
** Quality of life^2^**	76.71 (10.40) *n* = 19	81.00 (11.98) *n* = 12	80.58 (16.09) *n* = 12	81.57 (15.57) *n* = 10	85.13 (11.35) *n* = 8	84.38 (14.64) *n* = 8	4.86 *n* = 10
ERYT*^b^	81.36 (9.50) *n* = 11	86.00 (6.14) *n* = 7	87.14 (12.07) *n* = 7	89.80 (10.53) *n* = 5	91.87 (8.51) *n* = 5	91.60 (12.34) *n* = 5	8.44 *n* = 5
** Coord**	70.31 (8.26) *n* = 8	74.00 (15.26) *n* = 5	71.40 (17.69) *n* = 5	73.33 (16.27) *n* = 5	73.89 (1.84) *n* = 3	72.34 (9.87) *n* = 3	3.02 *n* = 5
** Sleep quality^3^**	7.93 (4.06) *n* = 15	7.75 (4.59) *n* = 12	6.45 (4.72) *n* = 12	6.50 (4.58) *n* = 10	6.75 (4.03) *n* = 8	7.25 (4.68) *n* = 8	−1.43 *n* = 10
** ERYT**	8.63 (3.46) *n* = 8	8.14 (4.22) *n* = 7	4.6 (4.17) *n* = 7	6.00 (2.30) *n* = 5	5.80 (2.95) *n* = 5	6.20 (4.02) *n* = 5	−2.63 *n* = 5
** Coord**	7.14 (4.81) *n* = 7	7.2 (5.54) *n* = 5	6.8 (5.81) *n* = 5	8.60 (5.55) *n* = 5	8.33 (5.77) *n* = 3	9.00 (6.08) *n* = 3	1.46 *n* = 5
** Pain severity^4^**	1.88 (1.21) *n* = 17	1.88 (1.87) *n* = 12	1.94 (1.76) *n* = 12	1.95 (1.75) *n* = 10	1.06 (1.02) *n* = 8	1.31 (1.74) *n* = 8	0.07 *n* = 10
** ERYT**	2.00 (1.19) *n* = 9	1.14 (1.27) *n* = 7	1.39 (1.31) *n* = 7	1.20 (1.54) *n* = 5	0.45 (0.41) *n* = 5	0.35 (0.78) *n* = 5	−0.80 *n* = 5
** Coord**	1.75 (1.30) *n* = 8	2.90 (2.22) *n* = 5	2.70 (2.17) *n* = 5	2.70 (1.76) *n* = 5	2.08 (0.88) *n* = 3	2.92 (1.77) *n* = 3	0.95 *n* = 5
**Pain interference^4^**	3.13 (2.55) *n* = 17	2.00 (2.05) *n* = 12	1.95 (1.82) *n* = 12	2.30 (2.40) *n* = 10	1.16 (1.77) *n* = 8	1.16 (1.86) *n* = 8	−0.83 *n* = 10
** ERYT**	3.24 (2.04) *n* = 9	1.35 (1.37) *n* = 7	1.39 (1.29) *n* = 7	1.09 (1.47) *n* = 5	0.40 (0.75) *n* = 5	0.37 (0.75) *n* = 5	−2.15*^c^ *n* = 5
** Coord**	3.00 (3.18) *n* = 8	2.9 (12.64) *n* = 5	2.74 (2.29) *n* = 5	3.51 (2.67) *n* = 5	2.43 (2.44) *n* = 3	2.48 (2.62) *n* = 3	0.51 *n* = 5
** Depression^5^**	8.53 (4.86) *n* = 17			5.80 (3.88) *n* = 10	5.08 (3.43) *n* = 8	5.75 (3.49) *n* = 8	−2.73 *n* = 10
** ERYT**	10.33 (5.07) *n* = 9			4.60 (2.19) *n* = 5	4.13 (2.21) *n* = 5	5.20 (2.28) *n* = 5	−5.73 *n* = 5
** Coord**	6.50 (3.96) *n* = 8			7.00 (5.05) *n* = 5	6.67 (5.03) *n* = 3	6.67 (5.51) *n* = 3	0.50 *n* = 5
** Anxiety^6^**	4.63 (2.87) *n* = 17			4.00 (2.58) *n* = 10	3.13 (2.36) *n* = 8	3.25 (2.38) *n* = 8	−0.63 *n* = 10
** ERYT**	5.25 (3.49) *n* = 9			2.80 (1.64) *n* = 5	1.80 (1.30) *n* = 5	3.20 (2.17) *n* = 5	−2.45 *n* = 5
** Coord**	4.00 (2.14) *n* = 8			5.20 (2.95) *n* = 5	5.33 (2.08) *n* = 3	3.34 (3.21) *n* = 3	1.20 *n* = 5
** Distress^7^**	5.00 (1.97) *n* = 17	5.17 (1.70) *n* = 12	5.33 (2.06) *n* = 12	5.50 (2.27) *n* = 10	5.75 (2.25) *n* = 8	5.57 (2.43) *n* = 8	0.50 *n* = 10
** ERYT**	4.44 (1.94) *n* = 9	5.23 (2.14) *n* = 7	5.14 (2.27) *n* = 7	5.80 (2.39) *n* = 5	5.40 (2.51) *n* = 5	5.60 (2.61) *n* = 5	1.36 *n* = 5
** Coord**	5.63 (1.92) *n* = 8	5.00 (1.00) *n* = 5	5.60 (1.95) *n* = 5	5.20 (2.39) *n* = 5	6.33 (2.08) *n* = 3	6.00 (2.65) *n* = 3	−0.43 *n* = 5
** Arm mobility^8^**	8.56 (1.55) *n* = 16	8.73 (1.35) *n* = 11	8.73 (1.62) *n* = 11	9.00 (1.50) *n* = 9	8.86 (1.46) *n* = 7	9.00 (1.15) *n* = 7	0.44 *n* = 9
** ERYT**	8.38 (1.60) *n* = 8	8.83 (1.47) *n* = 6	8.83 (1.47) *n* = 6	9.50 (0.58)4 *n* = 5	9.50 (0.58) *n* = 4	9.50 (0.58) *n* = 4	1.13 *n* = 4
** Coord**	8.75 (1.58) n = 8	8.60 (1.34) *n* = 5	8.60 (1.95) *n* = 5	8.60 (1.95) *n*=	8.00 (2.00) *n* = 3	8.34 (1.53) *n* = 3	−0.15 *n* = 3

Abbreviations: ERYT: Eurythmy therapy; Coord: CoordiFit. (1) Fatigue: Fatigue subscale of the Functional Assessment of Chronic Illness Therapy (FACIT-F)^20^ (range: 0-52; higher scores = less fatigue). (2) Quality of life: General score of the Functional Assessment of Cancer Therapy^21^^,^^22^ (range: 0-108; higher scores = higher quality of life). (3) Sleep Quality: Pittsburgh Sleep Quality Index^23^^,^^24^ (range: 0-21; higher scores = lower sleep quality). (4) Pain: Brief Pain Inventory^25^^,^^26^ (range: 0-10; higher scores = higher pain levels). (5) Depression: Patient Health Questionnaire-9^27–29^ (range: 0-27; higher scores = higher depression levels). (6) Anxiety: General Anxiety Disorder-7^27–29^ (range: 0-21; higher scores = higher anxiety levels). (7) Distress: National Comprehensive Cancer Network Distress Thermometer^30^ (range: 0-10; higher scores = greater distress). (8) Arm mobility rating (range: 0-10; higher scores = higher arm mobility). Depression and anxiety were measured only at baseline, intervention completion, and at both follow-ups.

a Cancer-related fatigue improved significantly in both groups, *P* = .006.

b Quality of life was higher in the ERYT group compared to the CoordiFit group, *P* = .028.

c Pain interference improved significantly in the ERYT group but not in the CoordiFit group, *P* = .022.

### Serious adverse events

There were no serious adverse events (SAEs) related to the interventions in this study. SAEs unrelated to the interventions that occurred during the study’s timeframe involved two participant deaths, one participant reporting new painful metastasis (gr. 3), and one participant reporting a decline in overall health (gr. 4).

## Discussion

Cancer-related fatigue is among the most taxing symptoms in breast cancer patients,^25^ significantly impacting their quality of life including daily activities, work, and social life.^31–35^ In patients with metastatic disease, the prevalence of cancer-related fatigue exceeds 75%^36^^,^^37^ and it often persists for years, even after successful cancer treatment.^38^ Currently, there is no gold standard for the treatment of cancer-related fatigue.^38^^,^^39^ Guidelines and meta-analyses report evidence that some degree of physical activity may relieve cancer-related fatigue.^36^^,^^40–42^ However, due to metastases or other comorbid illnesses, many types of physical exercises may be untenable for breast cancer patients.^36^ Increasing studies suggest mindful movement techniques such as Tai Chi or Yoga as a promising approach for reducing cancer-related fatigue,^18^^,^^36^^,^^38^^,^^39^^,^^43–45^ but research in this context is still emerging.^46^ We conducted a randomized controlled, open-label, two-arm, multi-center superiority trial to investigate the effect of ERYT (see description above) on fatigue in metastatic breast cancer patients, as compared to slow-paced physical exercises (CoordiFit). However, the trial had to be terminated before completion due to significant recruitment challenges leading to low enrolment. Nonetheless, very much in the spirit of *The Oncologist’*s *Clinical Trial Results* section, we consider it essential to make the trial outcomes publicly available in spite of the low enrolment, for it to serve future research as a basis and to safeguard fairness to the oncology patients who have participated in this study to advance scientific knowledge on treatment options that may benefit future patients.

We hypothesized that the ERYT intervention would be superior in reducing cancer-related fatigue compared to CoordiFit. A double-blind randomized controlled trial with 87 fatigued breast cancer survivors compared a meditative movement practice based on Qigong/Tai Chi with a sham Qigong that utilized similar movements to parallel the level of physical exertion, but without any meditative aspects. The study found that fatigue decreased significantly in the Qigong/Tai Chi group compared to the sham Qigong group after 12-weeks of intervention, which continued to hold true after a 3 months follow-up period.^47^ Another study compared aerobic exercises with aerobic plus Yoga in a sample of 52 breast cancer survivors.^48^ They found that although functional capacity, peripheral muscle strength, and quality of life improved significantly in both groups, a significant reduction in fatigue was observed only among the participants that had also practiced Yoga. Finally, a study with 274 participants suffering from chronic low back pain compared three different movement-based interventions in terms of their mental health effects, namely, standard physiotherapy, Yoga, and ERYT, the latter two involving a mindfulness aspect, in contrast to the former. The study found significant improvements on various mental health indicators for Yoga and ERYT, but not for the physiotherapy group.^15^

To test our hypothesis, we conducted two-way mixed ANOVA (time × group) models comparing completion with baseline scores. While we found a significant main effect of the interventions across time *F*(1,8) = 14.176, *P* = .006 (effect size $\eta_{G}^{2}=0.55$; power: 86%), with cancer-related fatigue decreasing significantly at the end of the intervention compared to baseline, this improvement was equally present in both groups, meaning that, contrary to our expectation, there was no interaction effect (*F*(1,8) = 0.239, *P* = .638, $\eta_{G}^{2}=0.02$; power: 7%). Further, there was no main effect for group (*F*(1,8) = 3.036, *P* = .120, $\eta_{G}^{2}=0.11$; power: 16%).

Of the secondary endpoints, two-way mixed ANOVAs comparing baseline with completion scores showed a significantly higher quality of life in the ERYT versus the CoordiFit group (main effect group: *F*(1,8) = 7.179, *P* = .028, $\eta_{G}^{2}=0.35$; power: 53%). Further, pain interference (as per Brief Pain Inventory scale the extent to which pain has interfered with daily activities, mood, relationships, etc.) significantly improved over time in the ERYT group, while it did not change over time in the CoordiFit group (interaction effect: *F*(1,8) = 7.977, *P* = .022, $\eta_{G}^{2}=0.08$; power: 13%). There were no significant results for the remaining secondary endpoints.

Our data could not confirm the superiority of ERYT to slow-paced physical exercise for the reduction of cancer-related fatigue, although statistical power was low due to the small sample size. The reported findings must hence be interpreted with caution. Nonetheless, the result that movement-based interventions in general may reduce cancer-related fatigue aligns with previous clinical studies^42^^,^^49–51^ as well as with the few studies available regarding ERYT. In conjunction with the latter, a controlled trial with 68 healthy adults found 10 h of ERYT over the course of six weeks, delivered in a group setting, significantly reduced fatigue.^6^ In an observational study with 125 subjects diagnosed with cancer, cancer-related fatigue was significantly reduced after an 8-weeks long intervention that included ERYT and other mindfulness-related exercises (group setting, online delivery).^7^ In a pragmatic comprehensive cohort study, a multimodal movement therapy that included ERYT significantly improved fatigue in breast cancer patients with chronic cancer-related fatigue.^8^ Finally, in a partially randomized controlled study in which breast cancer survivors with cancer-related fatigue were treated over 10 weeks, a multimodal program including ERYT as well as psychoeducational and art therapy was superior to an aerobics intervention in reducing fatigue, which held true also 4-years post treatment.^9^ Further, research suggests that ERYT may aid to reduce stress in oncology patients and in healthy individuals,^7^^,^^10–12^ and has also been associated with improvements in sleep quality, anxiety, and overall quality of life,^7–9^^,^^13–17^ which is however also true for other movement-based interventions.^52^^,^^53^

**Figure 1.**
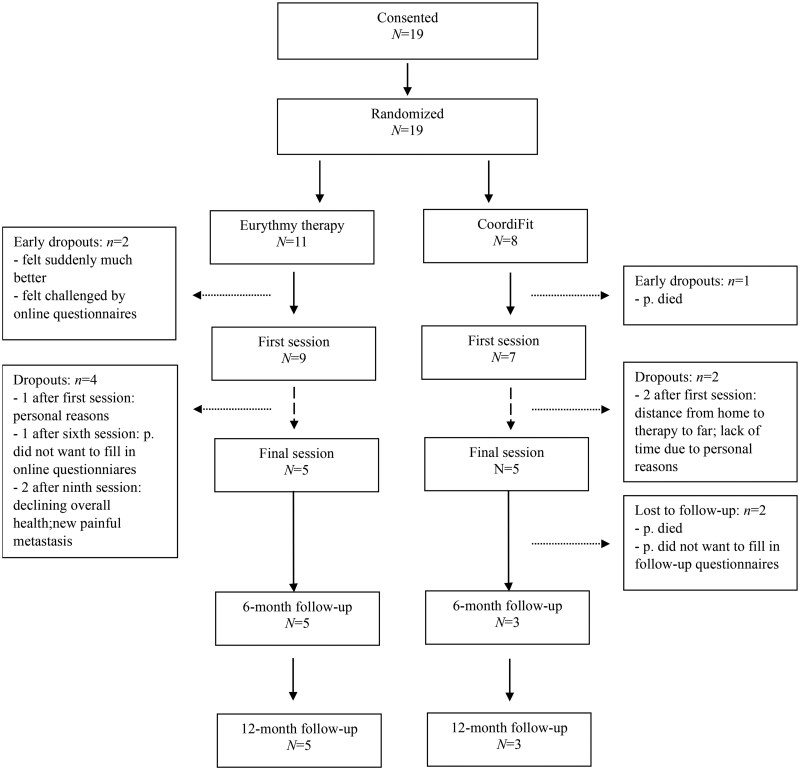
Flowchart of participant retention. Overall, *n *= 16 participants completed at least one intervention session (*m *= 9.6 sessions, range 1-13). Among the *n *= 10 participants who completed the full intervention, the mean number of sessions attended was 12 (range 8-13). Compliance rate for the home practice of exercises taught during the sessions was *m *= 1.58 h per week across the 20-weeks intervention (intervention completers).

The study had several limitations. As mentioned before, the trial had to be terminated before its completion due to insufficient enrolment. A number of factors contributed to this; the COVID-19 pandemic (March 2020-May 2023 as per World Health Organization^54^) caused significant disruption to the assessment and recruitment of eligible participants, as it did for other oncology trials.^55–57^ National restrictions to contain COVID-19 outbreaks, pressure on the healthcare system (and hence our study centers and its staff), as well as an understandable reluctance among cancer patients to participate in the trial given potential risks associated with in-person meetings during the pandemic years^58^ led to low rates of recruitment and participation at all study centers. Interestingly, even once the pandemic impact had lessened, study centers continued to show exceedingly low recruitment rates. A possible reason for this could be the comparatively low per patient fee that was offered to the participating centers (CHF 150.- per enrolled patient) due to limited study budget, which may have resulted in low incentivation and potentially led centers to favor studies with higher patient fee capacity (particularly if industry sponsored, such fees can amount to several thousand CHF in Switzerland).^59–61^ Since extensive efforts to increase enrolment did not bear fruit, trial recruitment was closed in February 2024. In Switzerland, around 25% of randomized clinical trials experience premature discontinuation, primarily due to difficulties in patient recruitment.^62^ An additional limitation was the lack of a nonactive control group to estimate effects attributable to the passage of time. Further, the study showed relatively high dropout (see Figure 1), which is however common for studies with metastatic cancer patients. The main reasons for dropout were factors directly associated with metastatic cancer. Additionally, some participants felt challenged by the completion of the questionnaires. Future studies should implement shortened questionnaire versions wherever possible and could consider delivering the survey in an interview format to facilitate more ease of application. One participant who dropped out relatively early did so due to the geographic distance to reach the movement-based therapy, a limitation which was subsequently addressed by providing therapist options in greater proximity to the centers.

**Figure 2.**
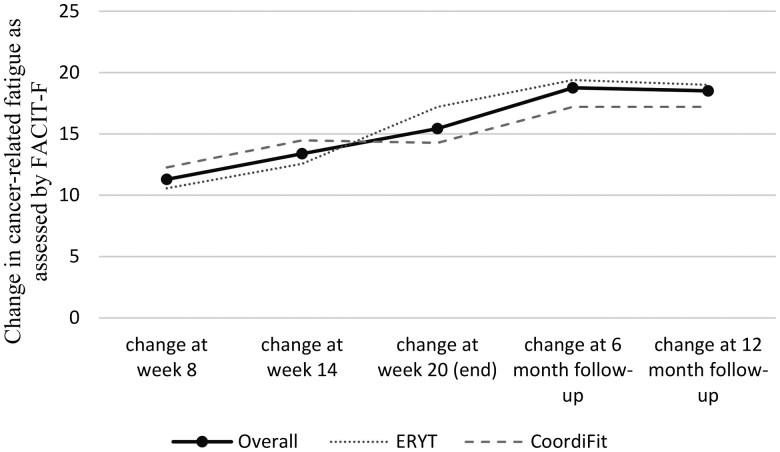
FACIT-F Fatigue change scores from baseline. FACIT-F: Functional Assessment of Chronic Illness Therapy—Fatigue subscale^20^; ERYT: Eurythmy therapy.

**Figure 3.**
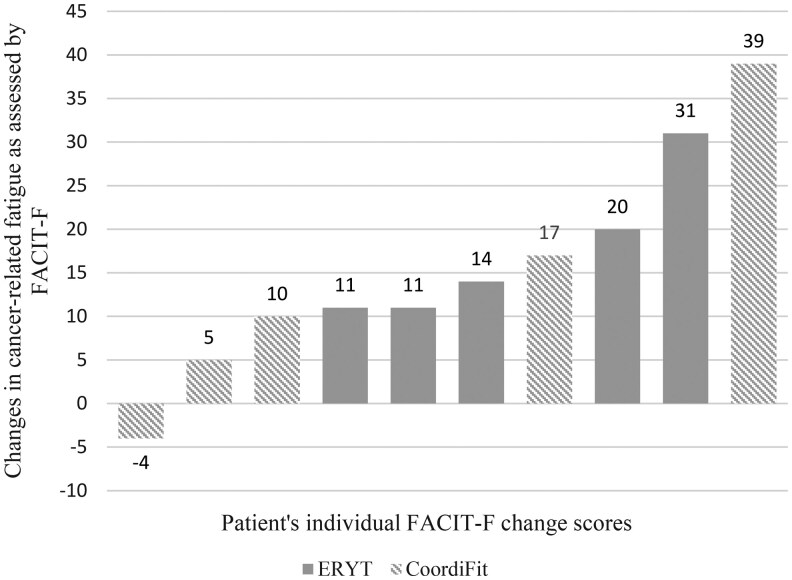
Change in fatigue per participant from baseline to intervention completion. The validated minimal clinically important difference for the fatigue subscale of the Functional Assessment of Chronic Illness Therapy (FACIT-F)^20^ is defined as a change of 3-4 points.^22^ Hence, 9 of the 10 completers showed a clinically important improvement in cancer-related fatigue; ERYT: Eurythmy therapy.

The study also has several strengths. It is the first clinical trial comparing ERYT with slow-movement physical exercises in view of cancer-related fatigue. We tested a nonpharmacological and safe intervention for an indication for which effective treatments are not yet available, but which is profoundly debilitating and highly prevalent among breast cancer patients. Further, the study employed an active control group to test the effects of ERYT, thereby controlling for differential impacts between specific therapists. Finally, participants reported satisfaction with the treatment, and the therapeutic staff that delivered the interventions remained motivated and engaged in the study despite the significant recruitment challenges. Based on the current findings, we would advise future designs to include a nonactive control group as a third arm to rule out effects of cancer-related fatigue improvement due to the passage of time alone, and ideally investigate a range of movement-based interventions for cancer-related fatigue in breast cancer patients to find out if any of them are superior. Both of these pathways, however, imply more complex, and therefore, costly designs, which is a relevant consideration in itself. Clearly, improved grant opportunities and funding for such studies will be needed to advance treatment options for cancer-related fatigue.

## Data Availability

The data underlying this article cannot be shared publicly to protect the privacy of individuals that participated in the study. The data will be shared on reasonable request to the corresponding author.
